# Measuring patient perceptions about osteoporosis pharmacotherapy

**DOI:** 10.1186/1756-0500-2-133

**Published:** 2009-07-14

**Authors:** Suzanne M Cadarette, Monique AM Gignac, Susan B Jaglal, Dorcas E Beaton, Gillian A Hawker

**Affiliations:** 1 Leslie Dan Faculty of Pharmacy, University of Toronto, Toronto, Canada; 2 Department of Health Policy, Management and Evaluation, University of Toronto, Toronto, Canada; 3 Division of Outcomes and Population Health, University Health Network, Toronto, Canada; 4 Department of Public Health Sciences, University of Toronto, Toronto, Canada; 5 Department of Physical Therapy, University of Toronto, Toronto, Canada; 6 Osteoporosis Research Program, Women's College Hospital, Toronto, Canada; 7 Institute for Work and Health, Toronto, Canada; 8 Mobility Program Clinical Research Unit, St. Michael's Hospital, Toronto, Canada; 9 Division of Rheumatology, Women's College Hospital, Toronto, Canada

## Abstract

**Background:**

Adherence to osteoporosis pharmacotherapy is poor, and linked with patient perceptions of the benefits of, and barriers to taking these treatments. To better understand the association between patient perceptions and osteoporosis pharmacotherapy, we generated thirteen items that may tap into patient perceptions about the benefits of, and barriers to osteoporosis treatment; and included these items as part of a standardized telephone interview of women aged 65–90 years (n = 871). The purpose of this paper is to report the psychometric evaluation of our scale.

**Findings:**

Upon detailed analysis, six of the thirteen items were omitted: four redundant, one did not correlate well with any other item and one factorial complex. From the remaining seven items, two distinct unidimensional domains emerged (variance explained = 78%). Internal consistency of the 5-item osteoporosis drug treatment benefits domain was good (Cronbach's alpha = 0.88), and was supported by construct validity; women reporting a physician-diagnosis or taking osteoporosis pharmacotherapy had higher osteoporosis treatment benefit scores compared to those reporting no osteoporosis diagnosis or treatment respectively. Because only two items were identified as tapping into treatment barriers, we recommend they each be used as a separate item assessing potential barriers to adherence to osteoporosis pharmacotherapy, rather than combined into a single scale.

**Conclusion:**

The 5-item osteoporosis drug treatment benefits scale may be useful to examine perceptions about the benefits of osteoporosis pharmacotherapy. Further research is needed to develop scales that adequately measure perceived barriers to osteoporosis pharmacotherapy.

## Background

Osteoporosis is a major public health problem resulting in considerable fracture-related morbidity [1,2]. Current therapies for osteoporosis maintain or improve bone density by affecting bone remodelling, and thereby reduce fracture risk [3]. However, rates of prescribing among those who would benefit, and adherence to therapy in those prescribed, are suboptimal. For example, fewer than half of fracture patients are treated to reduce the risk of recurrent fractures, and of those who initiate osteoporosis pharmacotherapy, more than 40% discontinue treatment within the year following initiation [4,5]. The Health Belief Model hypothesizes that individuals will not seek preventive care unless they perceive disease to be threatening, believe that health maintenance is efficacious (benefits), find barriers to care minimal, and receive cues to action [6]. Based on this model, a patient at high risk for fracture would not begin treatment, or adhere to pharmacotherapy, unless they believe the benefits of treatment outweigh its barriers. Consistent with this model, we recently found that patient perceptions of the benefits of pharmacotherapy were independently correlated with osteoporosis treatment [7]. The purpose of this paper is to report our psychometric evaluation of the scale used to examine patient perceptions of osteoporosis treatment benefits and barriers, so that others may benefit from our experience in scale development, and to facilitate future research examining patient perceptions about osteoporosis pharmacotherapy.

## Methods

Details of study recruitment have been previously published [8,9]. In brief, we sampled 1,500 women (750 from each of two regions) from a list of residents who had completed a short screener questionnaire about arthritis [10], for a new study focused on osteoporosis management. Community-dwelling women aged 65–90 years, residing within the two sampled regions, and able to complete the telephone interview were eligible. Data collection was completed by standardized telephone interview in 2003 and 2004. The questionnaire included thirteen items that we generated to measure patient perceptions about the benefits of, and barriers to osteoporosis pharmacotherapy. We generated the thirteen items using a similar format (item wording and response structure) to the Osteoporosis Health Beliefs Scale, that was also included as part of the standardized interview. The Osteoporosis Health Beliefs Scale was developed based on the Health Belief Model to measure six osteoporosis-related health beliefs: susceptibility, severity, exercise benefits, exercise barriers, calcium benefits and calcium barriers; and general health motivation [11,12]. Each item has a 5-point response option from strongly disagree to strongly agree. In generating our thirteen items, we were careful to include questions about "osteoporosis" separately from "broken bones" to differentiate between respondents who understand that osteoporosis is about brittle bones, from those who may not (e.g., confuse osteoporosis with osteoarthritis).

### Statistical Analysis

Measures of central tendency and response distribution were summarized for each of the thirteen items generated. Items to which over 80% of participants either agreed (strongly agree and agree) or disagreed (strongly disagree and disagree) were flagged as having poor discriminative ability [13]. Inter-item correlations were examined using polychoric correlation coefficients in PRELIS 2.72 (Scientific Software International, Lincolnwood, IL, USA). Pairs of items with a correlation of 0.90 or higher were considered redundant. Redundant items do not contribute additional information to the underlying construct being measured, yet add to respondent burden. We thus omitted redundant items to improve the utility of our scale for future use. After excluding redundant items, scale structure was evaluated by exploratory factor analysis using polychoric correlation and asymptotic covariance matrices in LISREL 8.72 (Scientific Software International, Lincolnwood, IL, USA). Our goal was to identify distinct (unidimensional) domains measuring patient perceptions about osteoporosis pharmacotherapy. We therefore excluded complex loading items. Kaiser-Guttman criterion of eigenvalues ≥ 1.0, Cattell's scree plots [14], and interpretability were used in deciding upon the number of factors to retain [15]. Factor loadings ≥ 0.4 were considered important and loadings ≥ 0.5 significant [16]. Final factor loadings were determined by orthogonal rotation using the normalized Varimax procedure [17]. The internal consistency of each domain was tested using Cronbach's alpha coefficients [15,18] calculated in SAS 8.02 (SAS, Cary, NC, USA). We calculated summative scores for each domain that demonstrated adequate internal consistency by adding the responses to each item identified by factor analysis.

As a sensitivity analysis, we randomly divided our sample in two, and tested scale structure first using exploratory factor analysis, and then using confirmatory factor analysis. Given that responses were not normally distributed across response options, we focused on the standardized root mean square residual (values < 0.1 being favourable), and the non-normed fit index (values > 0.9 recommended) in evaluating the performance of scale structure based on confirmatory factor analysis [16,19].

We tested construct validity of raw domain scores by hypothesized relationships using unpaired t tests or ANOVAs. We specifically hypothesized that osteoporosis drug treatment benefit scores would be higher among women reporting physician-diagnosed osteoporosis (versus not) and those currently taking osteoporosis pharmacotherapy (bisphosphonate, calcitonin and/or raloxifene), compared to past use or no osteoporosis drug exposure.

## Results

A total of 871 women participated by responding to the standardized telephone interview (participation rate = 84%, response rate = 72%). The mean and median age of study participants was 75 years (SD = 6.1). Participants were similar to nonresponders based on physician-diagnosed osteoporosis, fracture history and treatment with etidronate and hormone therapy, determined by self-report in 1995 and 1997 [20], Table 1. Participants were also similar in age (as of May 2003) to women refusing to participate (mean age = 76 years), but significantly younger than ineligible women (mean age = 78 years) and those not contacted (mean age = 77 years).

**Table 1:** Characteristics of study responders and non-responders,* N = 1,500

	**Participant**		**Refused**		**Unable to reach**		**Ineligible**	
	(N = 871)		(N = 171)		(N = 174)^†^		(N = 284)	
	n	(%)^‡^	n	(%)^‡^	n	(%)^‡^	n	(%)^‡^
Physician-diagnosed osteoporosis	94	(13.1)	16	(11.3)	24	(17.0)	36	(15.0)
Fracture since age 40^§^	122	(14.6)	16	(9.8)	11	(6.9)	36	(13.4)
Etidronate use (ever)	12	(1.6)	1	(0.7)	2	(1.5)	8	(3.5)
Hormone therapy								
never	552	(68.9)	113	(73.4)	108	(72.5)	187	(73.6)
now	113	(14.1)	25	(16.2)	16	(10.7)	36	(14.2)
past	136	(17.0)	16	(10.4)	25	(16.8)	31	(12.2)

* Based on self-reported data collected between 1995 and 1997 [20].

^† ^Includes 150 for whom we were unable to locate [8], and 14 that we were unable to contact by the end of study recruitment in 2004.

^‡ ^Proportions adjusted for missing data.

^§ ^Wrist, arm, hip, rib, pelvis, or vertebrae; proportions statistically similar except for differences between those "unable to reach," compared to participants or ineligible subjects.

Table 2 lists the thirteen items generated to assess patient perceptions about osteoporosis pharmacotherapy, and summarizes the response distribution for each item. One item was missing data for one respondent. Two items (8 and 9) had poor discriminative ability with over 80% disagreeing with each statement. Items 2, 4, 5 and 6 were redundant (polychoric correlations > 0.9) asking similar questions about "feeling good" or if the participant would "consider" taking drug treatments to prevent osteoporosis or broken bones. In an effort to tap both feelings toward taking drugs to prevent osteoporosis and whether one would consider pharmacotherapy to prevent fractures, two of these items (2 and 6) were retained. The Pearson correlation coefficient between these two items was 0.79 (polychoric correlation coefficient = 0.91). Items 11, 12 and 13 were also redundant, and of the three, item 11 was retained (based on length and clarity). At the other extreme, no item correlated well with item 7 (Drug treatment for osteoporosis cost too much); its highest correlation was with item 10 (r = 0.33). This item was therefore removed from the scale.

**Table 2:** Univariate statistics for items generated to measure patient perceptions about osteoporosis pharmacotherapy, N = 871

		**Distribution**		**Response Frequency (%)**				
**Item**		**mean**	**SD**	**1**	**2**	**3**	**4**	**5**
1	Drug treatments can help to build strong bones	3.49	0.66	0.1	8.5	33.6	57.6	0.1
2	You would feel good about taking drug treatments to prevent osteoporosis	3.14	0.91	1.0	31.8	19.9	46.7	0.6
3	Drug treatments can cut down the chances of broken bones	3.45	0.67	0.2	9.2	35.9	54.3	0.3
4	You would feel good about taking drug treatments to prevent broken bones	3.14	0.92	1.3	31.9	19.3	47.0	0.6
5	You would consider taking drug treatments to prevent osteoporosis	3.31	0.88	0.5	26.1	16.3	56.6	0.6
6	You would consider taking drug treatments to prevent broken bones	3.31	0.88	0.5	25.9	16.3	56.7	0.6
7	Drug treatment for osteoporosis cost too much*	2.58	0.59	0.3	46.5	48.3	4.7	0.1
8^†^	You are taking too many* medications	2.17	0.72	10.4	71.3	9.6	8.5	0.1
9^†^	You have stomach problems that limit your ability to take drug treatment	2.13	0.47	0.7	89.9	4.9	4.4	0.1
10^‡^	Taking drug treatments would upset your every day routine	2.32	0.65	0.5	75.9	14.7	8.7	0.2
11	If your doctor advised you to, you would take drug treatments to prevent broken bones	3.64	0.62	0.2	6.3	23.2	69.5	0.8
12	If your doctor told you that you were at high risk, you would consider taking drug treatments to prevent osteoporosis	3.81	0.64	0.3	4.8	15.5	72.6	6.8
13	If your doctor told you that you were at high risk, you would consider taking drug treatments to prevent broken bones	3.81	0.64	0.3	4.6	15.8	72.4	6.8

1 = strongly disagree, 2 = disagree, 3 = neutral, 4 = agree, 5 = strongly agree.

* Underlined words may be value-laden or vague.

^† ^Item has poor discriminative ability, i.e., >80% of respondents disagree (disagree or strongly disagree) with the statement.

^‡ ^Data available for 870 participants.

After excluding four redundant items (4, 5, 12 and 13) and item 7 that did not correlate well with any other item, the remaining eight items were submitted to exploratory factor analysis resulting in a 2-factor solution. However, item 10 (Taking drug treatments would upset your every day routine) demonstrated complex factor loading. Indeed, weekly bisphosphonates have been available in Canada since July 2002, calling the generalizability of the item into question. Therefore, item 10 was removed from the scale. New evidence has also found that weekly dosing of bisphosphonates after hip fracture in Pennsylvania increased from 26% in 2000 (when weekly regimen first available in the United States), to 100% in 2004 [21]. Subsequent factor analysis resulted in a 2-factor uncorrelated solution, Table 3. Domains were labeled based on the content of their items as: osteoporosis drug treatment benefits (57% variance explained, 5 items) and drug treatment barriers (21% variance explained, 2 items). Similar results were observed in sensitivity analyses. Among the 436 randomly allocated to exploratory factor analysis, we identified the same 2-factor uncorrelated solution (79% variance explained, 57% benefits and 22% barriers). Scale structure was further supported by confirmatory factor analysis among the remaining 435, with standardized root mean square residual of 0.08, and non-normed fit index of 0.98.

**Table 3:** Factor loadings for the osteoporosis drug treatment benefits and drug treatment barriers sub-scales, N = 871

		Factor Loadings*		
**Items**		**Benefits**	**Barriers**	**Unique Variance**
**Osteoporosis drug treatment benefits**†				
1	Drug treatments can help to build strong bones	**0.80**	0.07	0.36
2	You would feel good about taking drug treatments to prevent osteoporosis	**0.94**	-0.06	0.11
3	Drug treatments can cut down the chances of broken bones	**0.87**	0.10	0.25
6	You would consider taking drug treatments to prevent broken bones	**0.90**	-0.06	0.17
11	If your doctor advised you to, you would take drug treatments to prevent broken bones	**0.78**	-0.12	0.36
				
**Drug treatment barriers**†				
8^‡^	You are taking too many medications	-0.12	**0.85**	0.25
9^‡^	You have stomach problems that limit your ability to take drug treatment	0.14	**0.56**	0.69

*Varimax solutions calculated using polychoric correlation and asymptotic covariance matrices, explains 78.2% of model variance (benefits: 57.2%, barriers: 21.0%).

^† ^Sub-scales labeled based on item content.

^‡ ^Items 8 and 9 reverse coded prior to factor analysis.

Internal consistency of the osteoporosis drug treatment benefits domain was high (Cronbach's alpha = 0.88). With only 2 items in the barriers domain, the internal consistency of the scale was low and we do not report the Cronbach's alpha coefficient. The polychoric correlation coefficient between the two items was 0.46. In addition, in terms of face validity, the items appear to tap unrelated constructs; one taps into subjective impression (too many medication), and the other assesses medical contraindications to therapy/other conditions that affect use of medications (limited due to stomach problems). For these collective reasons, construct validity testing was restricted to the "benefits" domain. As hypothesized, women reporting to have a physician-diagnosis of osteoporosis had higher osteoporosis drug treatment benefit scores compared with those reporting no diagnosis (18.5 vs. 16.6, p < 0.001). Similarly, women taking pharmacotherapy (bisphosphonates, calcitonin and/or raloxifene) had higher osteoporosis drug treatment benefits scores: 19.6 for current use compared with 16.5 among past users and 16.4 for never users (p < 0.001). Although some hypothesized differences were small in magnitude, collectively all of these data (factor analysis, reliability and validity) suggest that the osteoporosis drug treatment benefits scale measures perceived benefits of osteoporosis drug treatment.

## Discussion

Understanding patient perceptions about the benefits of, and barriers to taking medications may help to understand why those with clinical need for a preventive agent may not initiate therapy or do not adhere to long-term therapy. We generated a number of items to tap different aspects of osteoporosis drug treatment attitudes and perceptions. These included issues related to costs of treatment, side effects, prevention of broken bones, and the role of physicians in taking medication. From among these items, a factor emerged related to perceived benefits of osteoporosis pharmacotherapy in preventing broken bones. This factor had good internal consistency and some preliminary validity suggesting that it may be useful to include in studies that examine drug treatment adherence and osteoporosis management more generally.

Nonetheless, more research needs to be conducted to examine whether there are other dimensions that were not sufficiently captured by the thirteen items that we generated. The benefits of pharmacotherapy examined in our scale focused on fracture prevention. Although this is directly in line with the Health Belief Model [6], suggesting that a woman will not take action unless she believes it to be efficacious, we cannot comment on whether there are other benefits that we did not consider or capture. In particular, we need to learn more about patient perceptions regarding the barriers to osteoporosis pharmacotherapy. Prior research has documented several potential barriers to adhering to osteoporosis pharmacotherapy, including: side effects (e.g., gastrointestinal tolerability), complexity of dosing regimen, patient safety concerns, inadequate patient knowledge, lack of noticeable symptoms or symptom improvements, patient preferences for medication use in general, patient-provider communication, patient involvement in treatment decision making and adequacy of treatment follow-up [22-24]. Our methodology in generating items is limited by not having tapped into all of the potential barriers to osteoporosis pharmacotherapy. Indeed, there are standard methods for generating items when designing multi-items scales. Different strategies may include one or more of the following: conducting focus groups, key informant interviews, clinical observation, theory, research and expert opinion [13,25]. Although our items are theory-driven (Health Belief Model), they do not cover the potential breadth of barriers to osteoporosis pharmacotherapy. It is therefore not surprising that we were unable to identify different domains tapping into specific barriers to osteoporosis treatment. Being faced with the desire to include patient perceptions regarding the benefits and barriers to osteoporosis pharmacotherapy, and the dilemma of not finding a validated scale at the start of data collection, we opted to modify existing items from the Osteoporosis Health Belief Scale. Although this strategy is not a robust means of creating multi-item scales, by working with a previously validated scale that examines osteoporosis health beliefs, we were able to develop a multi-item scale that measures patient perceptions regarding the benefits of osteoporosis pharmacotherapy.

## Conclusion

The 5-item osteoporosis drug treatment benefits scale has good psychometric properties among older women and may be useful in future research studies. However, further examination of the properties of the scale is recommended, and further work to identify and develop methods to measure the diverse barriers to osteoporosis pharmacotherapy is warranted.

## Competing interests

The authors declare that they have no competing interests.

## Authors' contributions

All authors contributed to study conception, design, and funding, as well as manuscript revision. SMC also contributed to data collection, completed statistical analyses, and prepared the manuscript for publication (drafted and revised). MAMG and DEB contributed to interpreting statistical analysis. All authors read and approved the final version of the manuscript.
